# Chinese Mobile Health APPs for Hypertension Management: A Systematic Evaluation of Usefulness

**DOI:** 10.1155/2018/7328274

**Published:** 2018-03-18

**Authors:** Jun Liang, Xiaojun He, Yuxi Jia, Wei Zhu, Jianbo Lei

**Affiliations:** ^1^IT Center, Second Affiliated Hospital, School of Medicine, Zhejiang University, Hangzhou, Zhejiang Province, China; ^2^College of Medical Technology, Zhejiang Chinese Medical University, Hangzhou, Zhejiang Province, China; ^3^Editorial Department of Chinese Journal of Emergency Medicine, Second Affiliated Hospital, School of Medicine, Zhejiang University, Hangzhou, Zhejiang Province, China; ^4^Department of Medical Informatics, School of Public Health, Jilin University, Changchun, Jilin Province, China; ^5^Provincial Key Cardiovascular Research Laboratory, Second Affiliated Hospital, School of Medicine, Zhejiang University, Hangzhou, Zhejiang Province, China; ^6^Center for Medical Informatics, Peking University, Beijing, China; ^7^School of Medical Informatics and Engineering, Southwest Medical University, Luzhou, Sichuan Province, China

## Abstract

**Objective:**

To analyze and compare the usefulness of hypertension management APPs released in the Chinese market; to understand the general situations, characteristics, problems, and trends in hypertension management mHealth APPs; and to identify the gaps between mainland China products and non-mainland China products with the aim to provide recommendations for developers in industry and assist hypertensive patients in selecting suitable APPs.

**Methods:**

The hypertension management APPs available by October 2016 in China were analyzed from the perspective of data items and function usefulness. Sample sets were determined through PRISMA. An evaluation item set was developed based on the usability framework of TURF and the *Chinese Guideline for the Management of Hypertension* and used to quantitatively analyze the functionalities and data items collected from the sample APPs from the perspective of designers, users, and activity models.

**Results:**

Among the 73 Chinese-supported APPs, none of the hypertension management APPs could fully cover the usefulness item set (mean = 37.4%). Regarding the use of mobile terminal hardware, only cameras and positioning sensors are commonly used in information collection. Regarding the data items and services provided, the most commonly collected data are “demographic information” (88% versus 100%) and “vital signs” (76% versus 100%), but APPs developed in mainland China and non-mainland China provided significantly different services and profit-making patterns. Regarding data security and privacy protection, the APPs from mainland China provided far lower usefulness (31% versus 56%).

**Conclusions:**

mHealth APPs can promptly and efficiently acquire sign-related data by improving the professionality and scientificity of data about healthy living habits. APPs also improve the preventive usefulness of the collected data and bring about new opportunities for the management and control of hypertension. Other important research trends include privacy protection and data security.

## 1. Introduction

The death risk of hypertension ranks first worldwide and is intensified with aging. Unfortunately, the “traditional passive medical mode” has failed to meet the demands for chronic disease health management of the current aging society in China. The *Report on the Status of Nutrition and Chronic Diseases of Chinese Residents (2015)* [1] states that by 2015, hypertension had affected 20% of the global population (above age 18), which was lower than the incidence among Chinese adults (33.5%), and in particular, the incidence rate among the 70–74 age group in China was up to 58.6%. The death rate of hypertension ranks first and is 2.6 times higher than the second highest “diabetes” [2]. In contrast, the awareness rate of hypertension by patients is only 30.5%, and the controlled rate is only 4.2% [2]. Unfortunately, routine hypertension management methods become less efficient, due to the lack of detailed management and the communication barrier between doctors and patients' family members. The *Healthy China 2020 Strategic Research Report* [3] shows that the medical treatment system has moved to “focusing on prevention and control, aiming to transform the medical mode.” Technically, the emergence of numerous “home blood pressure” mHealth APPs has introduced potential changes [4–6].

As a subdiscipline of eHealth, mHealth is a newly appearing health mode in recent years [7] and brings new opportunities and challenges to hypertension prevention. The explosive growth of the Internet economy and the reform of medical treatment systems have accelerated the growing mHealth market in China. The annual growth rate in 2015 was 49%, and the overall assessed value in 2017 is expected to reach 1.9 billion dollars [8].

As a major research target in the field of HCI, usability refers to how useful, usable, and satisfying a system is for the intended users to accomplish goals in the work domain by performing certain sequences of tasks [9]. TURF, as one of the major and mainstream methods for usability evaluation, is a theoretical framework that assesses the usability of EHRs from three dimensions, including usefulness, usability, and user satisfaction [9–11]. According to the definitions of TURF, the usability evaluation of eHealth products is divided into degree of inherent complexity (usefulness) and degree of exogenous complexity (usability and satisfaction) [10, 11]. Usefulness reflects the complexity of work tasks and the effectiveness of software. Usefulness can be assessed from the perspectives of services and functions of eHealth products under the valid user context [12]. TURF further defines “designer model” (a collection of services concretely implemented in each software), “user model” (a collection of unambiguous services demanded by users, corresponding to the core demands of key users, including expert consensuses, clinical guidelines, specification, and standards), and “activity model” (a collection of services practically used by users in a real working environment and determined through comparison, analysis, and collection of relevant literature and data) [13]. A higher matching degree among these three models indicates that the software services are more useful. The usefulness assessment does not necessarily depend on user tests.

Design and implementation of a mHealth APP is not just an IT project but a workflow activity and human-computer interaction engineering project [7]. However, the current studies were mostly focused on the characteristics and coverage of functionalities from the perspective of designers rather than users [14–16]. Few studies have been performed to evaluate the functionality of APPs in the context of user-meaningful operations. Furthermore, evaluating the completeness and effectiveness of data items is important because this is the foundation for subsequent data analysis and services [17].

### 1.1. Significance of This Study

Along with the special rectification by the China National Health and Family Planning Commission since May 2017 [18], the limitation that only chronic disease management paid services are available in the mHealth market will further stimulate explosive development of this segmented market. However, there is no quantitative assessment about the usefulness of hypertension management APP products in the Chinese market. The usefulness of an APP varies from person to person and is largely affected by the subjective initiative. In this work, using TURF-originated usability evaluation measures, we made a first attempt from the perspective of designer, user, and activity models to compare the data elements and service usefulness of mHealth APPs developed in mainland China and non-mainland China targeting hypertension management in the Chinese market and investigated the characteristics, problems, and trends. We aimed to identify the gaps in Chinese products from foreign products in terms of usefulness, which should help to solve problems during APP development in this field and provide users assistance in the selection of appropriate APPs. This is also the first quantitative study on the usefulness of mHealth products developed in mainland China. The useful degree evaluation template for hypertension management proposed here can be applied to the development and evaluation of efficient products targeting chronic disease management. We also aim to minimize the risks of relevant complications by using the services of standardized APPs to improve medical treatment efficiency and reduce costs. This work will help to build a comprehensive and personalized management system covering tens of millions of hypertensive patients in China.

## 2. Materials and Methods

To our knowledge, this is the first time that a TURF usability evaluation tool has been used to quantitatively analyze the usefulness of main functionalities and data items collected from hypertension APPs from the perspective of the designer, user, and activity models.

### 2.1. Software Information Sources and Searching Term Selection

After market investigation, we determined 2 information sources: the Android platform and the iOS platform, which account for 70% and 21%, respectively, of the Chinese smartphone market. These two platforms correspond to two official software markets: Google Play store and Apple APP store. Targeting the theme of hypertension prevention, we focused on the APP categories of Health & Fitness and Medical. Terms including “高血压” and “Hypertension” were used to search the official search engines in October 2016.

### 2.2. Selection of Target APPs

**Figure 1:**
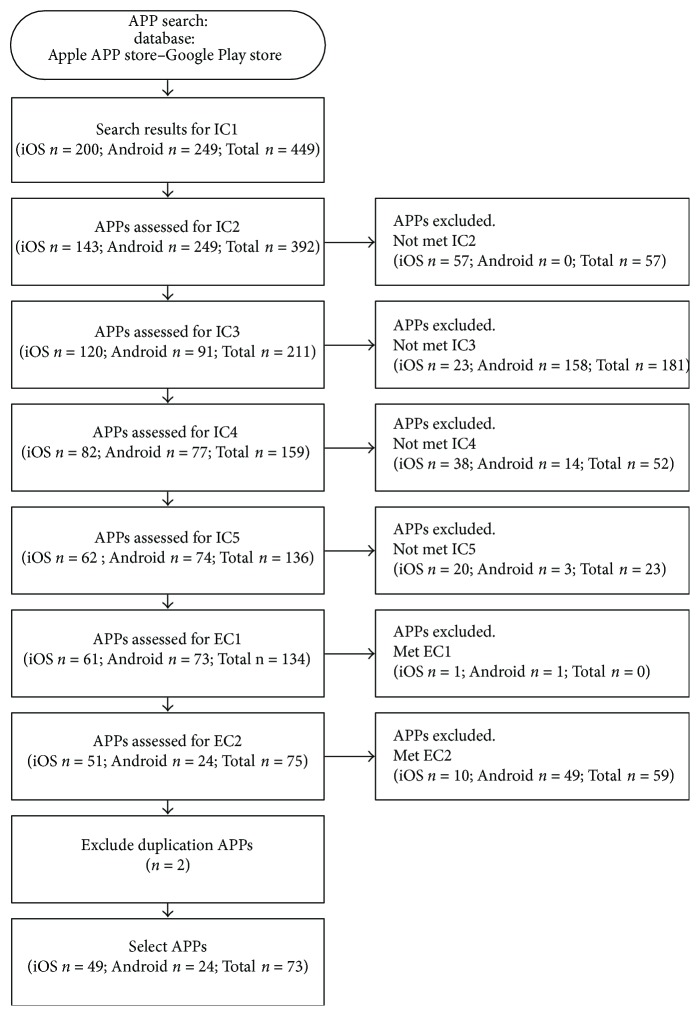
PRISMA flow diagram.

To select appropriate target APPs from all mHealth APPs for hypertension prevention, we used the Preferred Reporting Items for Systematic Reviews and Meta-Analysis (PRISMA) [19], which is widely applied in other eHealth studies, to define inclusion criteria and exclusion criteria (Table 1); namely, any target APP to be included should obey all inclusion criteria and exclusion criteria. The entire flowchart of APP selection is illustrated in Figure 1.

### 2.3. Development of TURF-Based Usefulness Evaluation Models

The TURF framework was used to evaluate the usefulness of the data element and services for the included APPs. According to the definitions of TURF, the usability of eHealth outputs is divided into degree of inherent complexity (usefulness) and degree of exogenous complexity. The former is quantified by “designer model,” “user model,” and “activity model” [12]. We hypothesized that for a perfect function design, the three above models should be equivalent. In reality, however, these three models are more or less different because they are all subjective, which makes it possible for product modification anyway.

### 2.4. Definition of Data Element Usefulness

As for the usefulness evaluation of data elements, we mapped the “designer model” into the data elements associated with hypertension management acquired and stored by each APP, so the “designer models” of APPs have different instances. The user model was mapped as follows: based on the *Basic framework and data standard of electronic health records* from Chinese residents [20], namely audited by the Chinese National Health and Family Planning Commission, to support efficient hypertension management and ensure that the data collection consensus acquired by information systems is the smallest. The activity model was mapped into the patient data associated with hypertension management measures defined by the “2010 Chinese guideline for the management of hypertension” [21] or the real medical treatment information that doctors should refer to when they provide a specific hypertensive patient with appropriate management and treatment services.

### 2.5. Definition of Service Usefulness Evaluation

To evaluate the usefulness of APP services, we mapped the designer model into the function set realized by each APP, so this APP had unique designer model instances. The service factors of mHealth [8, 22] are a generalized function template that describes the positions of mHealth APP services in the flow of continuous medical services. Under the context of hypertension management studies, we trimmed and customized this template using the principle of Population, Interventions, Professionals, Outcome and Health (PIPOH) [23] and obtained a service factor classification targeting the user model. This corresponds to the user model, namely the smallest function classification that is demanded by users of mHealth hypertension management APPs (Tables 2 and 3). Then, through an extensive literature review, we collected and analyzed the functions and behaviors of users during daily hypertension management, which correspond to the activity model. These concrete functions and behaviors include automatic or manual data processing; healthy living habits; acquisition of behavior-related knowledge; social communication, calendar-based event reminders, and remarks; time axis-based data processing and display; efficient communication in case of emergencies; and security and privacy protection in data backup and transmission.

### 2.6. Definition and Evaluation of Useful Degree Evaluation Item Template

Finally, according to the intersection between the user model and the activity model from the data elements and service set, we defined a usefulness evaluation item template involving 12 data items, 17 service evaluation options, and 9 safety and privacy indices (Table 4). The usability of concrete products in the data elements and service set was determined by assessing how many evaluation items in the user-defined template (corresponding to the intersection between the user model and the activity model) were covered by the APP (corresponding to the designer model).

## 3. Results

### 3.1. Basic Information of mHealth APPs

**Figure 2:**
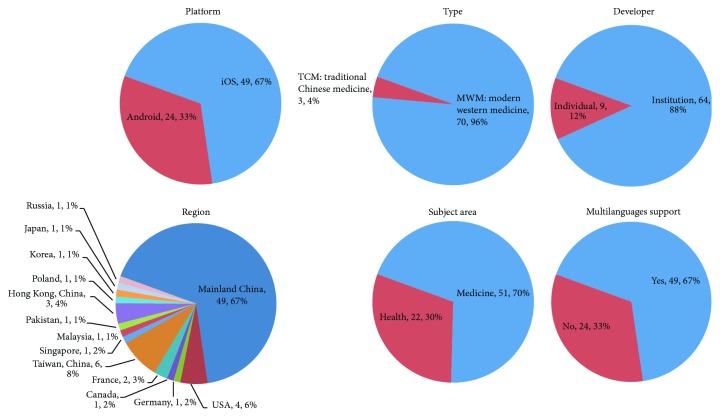
Distributions of the selected APPs by platform, type, developer, regions, subject area, and multilanguage support.

Mobile-end hypertension management is widely demanded. In this work, we selected 73 APPs (Table 5), including 49 iOS APPs and 24 Android APPs. These APPs were developed mostly by institutions (64 APPs) and a few by individuals (9 APPs). There are 51 medical APPs and 22 health APPs. Regarding the origins of developers, these APPs were developed in mainland China (49 APPs) and non-mainland China, including Chinese Taiwan (6 APPs), the United States (4 APPs), and other countries (e.g., France, Germany, Canada, Poland, Japan, South Korea, and Russia), indicating that demand came widely from three continents. Meanwhile, the multilanguage support rate of these APPs is up to 67%, which indirectly supports this viewpoint. Additionally, as a Chinese characteristic, three of the APPs support the management of hypertension from the perspective of traditional Chinese medicine (Figure 2).

### 3.2. Overall Usefulness Evaluation

**Figure 3:**
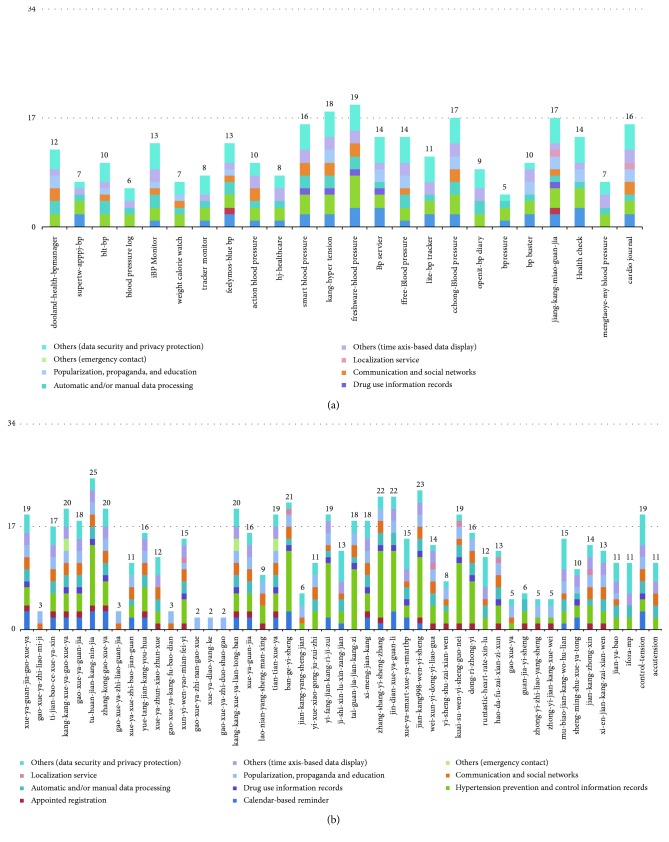
(a) Selected Android APP score classifications (24 APPs). (b) Selected iOS APP score classifications (49 APPs).

The useful degrees are highly specific among different APPs and generally are not high (mean = 37.4%). None of the APPs could cover 100% of the usefulness evaluation template. Among the Android APPs, the top three rankings by usefulness are “freshware-bloodpressure” (56%), “kang-hypertension” (53%), and “cchong-BloodPressure” and “jiang-kang-miao-guan-jia” (both 53%), and the last one is “bpressure” (15%). Among the iOS APPs, the top three rankings by usefulness are “tu-huan-jian-kang-nin-jia” (74%), “jian-kang998-wen-yi-sheng” (68%), and “zhang-shang-yi-sheng-zhang” and “jin-dian-xue-ya-guan-li” (both 65%), while the lowest are “gao-xue-ya-zhi-nan-gao-xue,” “xue-ya-diao-yang-ke,” and “gao-xue-ya-zhi-duo-shao-gao” (all 6%). In all, the iOS APPs have slightly higher useful degrees than the Android APPs (39% versus 32%), which is consistent with a previous study [14]. We think the differences may be attributed to the strict content auditing of Apple stores. The usefulness evaluations of APPs under different platforms are shown in Figure 3, where the *y*-axis is the number of items covered by an APP. Theoretically, one APP could cover up to all 34 usefulness measuring items.

### 3.3. Concrete Support Conditions of Data Elements and Services

**Figure 4:**
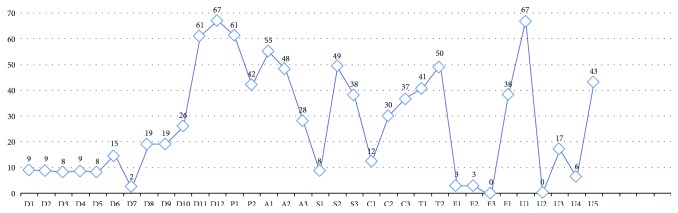
Item score classification. Notes: APP sample size = 73; score item size = 38. Calendar-based reminder (C1~C3); appointed registration (A3); automatic and/or manual data processing (P1, P2); localization service (S1); communication and social networks (S2, S3); popularization, propaganda, and education (A1, A2); others (emergency contact) (E1~E3); others (time axis-based data display) (T1, T2); others (data security and privacy protection) (F1, U1~U5); hypertension prevention and control information records (D1~D9, D11, D12); drug use information records (D10).

The supporting degrees of data elements and services are largely different among the APPs. As for single items, none of the items could be covered by all 73 APPs. In particular, the highest support degrees come from D12 and U1 (approximately 92%), which are both supported by 67 APPs, but the lowest come from E3 and U2 (both 0%). The real distribution of each data element or service is shown in Figure 4.

### 3.4. Comparison of Data Element and Service Usefulness between Mainland-Developed and Non-Mainland-Developed APPs

**Figure 5:**
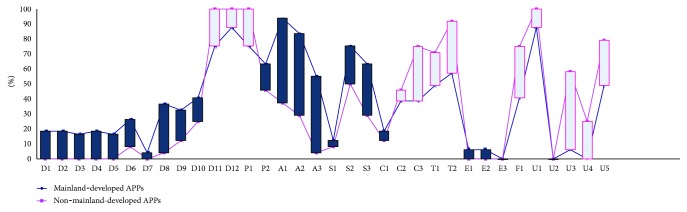
Comparison of data element and services between mainland-developed APPs and non-mainland-developed APPs. Notes: mainland-developed APP sample size = 49; non-mainland-developed APP sample size = 24; score item size = 38. Calendar-based reminder (C1~C3); appointed registration (A3); automatic and/or manual data processing (P1, P2); localization service (S1); communication and social networks (S2, S3); popularization, propaganda, and education (A1, A2); others (emergency contact) (E1~E3); others (time axis-based data display) (T1, T2); others (data security and privacy protection) (F1, U1~U5); hypertension prevention and control information records (D1~D9, D11, D12); drug use information records (D10).

Designers, users, and medical professionals from China and abroad have very different views about what functionalities should be contained and what data items should be collected from hypertension APPs. The two most commonly collected data elements for mainland-developed and non-mainland-developed APPs are “demographic information” (88% versus 100%, resp.) and “vital signs (e.g., height, weight, blood pressure, or heart rate)” (76% versus 100%, resp.), but the most commonly provided service is “promoting or self-carrying popularization and recommendations on health habits” (94%) and “patient data entry” (100%), respectively. Moreover, the mainland-developed APPs have a higher useful degree in data elements (33% versus 21%, resp.) and a lower useful degree in services (42% versus 43%, resp.), especially lower degrees in data display, system framework, security and privacy, and data transmission (31% versus 56%, resp.) (Figure 5).

## 4. Discussion

### 4.1. The Overall Usefulness of Hypertension Management mHealth APPs Is Generally Unsatisfactory

Hypertension management depends on the mobility, promptness, and barrier-free access of mobile devices and aims to customize professional mHealth APPs according to patient demands. However, the usefulness of such APPs is unsatisfactory and thus can be largely improved in the future (usefulness degree of neither type of APP exceeds 40%). Additionally, the accuracy of functions is controversial, and the functions are exaggerated. For instance, the Android APP “cchong-BloodPressure” states that users can collect body pulse data through the phone cam; its principle is to count pulses by periodically filming fingertip brightness to form RGB images. The heart rates acquired can only be references, but the APP claims to provide both systolic blood pressure and diastolic blood pressure, which is exaggerated and not science-based. We think these APPs do not meet the user demands for hypertension prevention and are unable to cover the majority of functions. In the future, more comprehensive and more professional APPs should be developed.

### 4.2. APPs Are Far from Embodying the Hardware Advantages of Smart Mobile Devices

The existing mHealth APPs targeting hypertension management have not been adjusted or optimized to the optimal use status of mobile devices. At present, the mHealth APP market in China is explosively growing due to the popularization of smartphones [29]; friendly human-phone interactive interfaces [30]; the convenient, prompt, and barrier-free access of mobile phones [31]; and the bonus of living habits brought by mobile-end E-commerce [32]. Although APP developers can use different sensors carried by mobile facilities, the commonly used device is only the cam; namely, P2 was supported by approximately 58% of APPs (42). In contrast, S1, another position-based sensor (GPS), was only supported by 11% of APPs (8).

### 4.3. Differences in Data Elements and Services Compared between Mainland-Developed and Non-Mainland-Developed APPs

The philosophies about hypertension and its treatment differ in the medical field and among the public, which probably has led to the differences in data element collection and support services among APPs. Chinese researchers think hypertension is a disease due to living habits [21]. APPs can be used to establish a planned, targeted, and evaluable program-like health education mode; therefore, the philosophy of healthy living can be introduced into health education, which makes information on health living habits as pushed more scientific and practical. On the contrary, overseas researchers generally believe hypertension is a disease with elevated blood pressure [33]. Thus, one major way to provide auxiliary treatment is to collect information about vital signs, such as weight, blood pressure, and heart rate. Moreover, as for the overall usefulness of data elements, mainland-developed software surpasses non-mainland-developed software (33% versus 21%, resp.), which implies that mainland APP developers are better at understanding users' actual demands for hypertension management.

### 4.4. Limitations of Mainland-Developed APPs in Information Security and Privacy Protection

Non-mainland-developed APPs largely differ from mainland-developed APPs in terms of information security and privacy protection. As for service usefulness, the mainland-developed APPs are slightly lower than the non-mainland-developed APPs (42% versus 43%, resp.) but especially in information security, privacy protection, and data display (31% versus 56%, resp.). At the level of either market self-discipline or governmental regulations, China has no concrete practical supervision and management measure targeting the information security of mHealth APPs. On the one hand, the majority of APPs do not release, on the user protocol or the supportive websites, any declaration about user data security or privacy protection, which is a hidden risk when individuals or institutions, either informed or not informed, utilize user privacy information to acquire economic benefits. On the other hand, the Chinese State Council released 11 official documents between 2013 and 2015 [14] that indirectly affected the information security and privacy protection of mHealth APPs, but the Chinese government has neither provided clear definition about the attributive right of medical treatment data nor issued any professional or direct supervision and management act like the American Health Insurance Portability and Accountability Act [34] or 45 CFR 170.314 [35]. At the technical level, some APPs (e.g., U1, U3, and U4) support certain security mechanisms, but the proportion is very low, and the nature of mobile device subjects' data storage put them at an extremely high risk of breach.

### 4.5. Differences in Profit-Making Patterns between Mainland-Developed and Non-Mainland-Developed mHealth APPs

Mainland-developed APPs and non-mainland-developed APPs largely differ in profit-making modes. Non-mainland-developed mHealth APPs almost all focus on disease monitoring and recording. The medical systems of Western countries, Hong Kong, Taiwan, and Macau, the global market, and the governmental laws have made drug sales not the key-profiting factor of APP suppliers but improvement of the profitability of monitoring facilities. In the above regions, the hierarchical diagnosis and treatment systems are complete, so the APP-recorded daily sign information helps general practitioners to continuously and consistently treat/manage hypertensive patients. In contrast, the mainland-developed mHealth APPs mostly focus on the provision of information for users. Specifically, the supportive rates of A1, A2, and A3 are up to 94%, 84% and 55%, respectively, and their profit-making modes are more diversified, including ads, service charge for rapidly and efficiently acquiring high-quality medical resources, and sales of monitoring devices. These differences are mainly attributed to the economy, population, and medical systems. Statistics in 2015 show that mainland China was the second largest economy with GDP up to 10,140 billion dollars [36], but its population was also the largest [37]. Even worse, its medical expenditure was only 5.95% of GDP [38], and the per capita medical cost was 438 US dollars or only 1/22 that of the US [39]. Moreover, the medical resources were very unbalanced between urban and rural areas, as the medical expenditure in urban areas was 2.52 times that in rural areas [40]. Due to a lack of a comprehensive hierarchical diagnosis and treatment system, rural patients do not have access to locally qualified medical resources and instead swarm into cities and compete with urban patients for limited special medical resources. On the contrary, owing to their functional characteristics, mainland-made mHealth APPs utilize the prevalence, convenience, and instant barrier-free access of smart mobile devices in China, which partially facilitate the acquisition of health information and medical services by this segment of patients and at least improve their chances to acquire services from doctors.

## 5. Suggestions

### 5.1. Create Professional mHealth Data Mining and Analysis

A professional mHealth APP should be decided by user retention and loyalty. If an APP only simply acquires, organizes, and displays data, such as the simple teaching of health-related information as popularization, propaganda, and education (A1, A2), then its attraction to users will gradually decrease. To continually expand the user group, APP need support from relevant data analysis and mining backend platforms that professionally analyze the data uploaded by users and convey it to users in an easy and understandable way. In response to the health problems identified from data analysis, the backend platforms can give reasonable and effective recommendations. When a user experiences an improvement in health status, he/she spontaneously has retention and thereby loyalty.

### 5.2. Improve User Privacy Protection Mechanisms

Owing to the uniqueness of the “Internet + healthcare” mode, user information becomes more concentrated and accessible, so there might be bugs in any link between online and offline, which harbor the risk of leaking user identity information and health data. Thus, it is urgent to build third-party Internet health information management platforms that rely on the industry association and improve user privacy protection mechanisms. Users are suggested to strengthen their consciousness of privacy protection and their sense of data possession and autonomous acquisition rights. Medical staff is recommended to sufficiently respect users' right of informed consent and not to use or leak user private information. Technically, the construction of third-party health information management platforms should be based on privacy protection systems with controlled data access; through the access-right restriction, it is ensured the accessing subject (medical staff) only reasonably and legally uses the accessed object (data deposited in the mHealth APP). Moreover, relevant functional departments and the industrial association are recommended to enhance supervision and management over the participating subject.

### 5.3. Improve Relevant Laws and Regulations

The legal subjects of mHealth include medical staff, users, medical institutions, and APP service providers, but their definitions and specifications in relevant Chinese laws are ambiguous. However, relevant laws and regulations should be improved as APP users are going to more frequently use Internet health and mobile medical intelligent devices.

### 5.4. Enhance Supervision and Management over mHealth APP and Wearable Medical Equipment

The mHealth APP and wearable medical equipment are supplementary to each other under the age of big data. The supplement of high-precision wearable equipment to the mHealth APP will largely promote the realization of targeted and individualized medical treatment, especially for chronic diseases such as high blood pressure. Thus, the industrial association is recommended to establish inclusion criteria for mHealth APPs and wearable medical equipment, which should ensure security, practicability, and effectiveness but not restrict the development and innovation in this industry. When using a mHealth APP, users usually first find out the quality defects and risks. Thus, it is recommended to build a supervision feedback channel, so users will become the main force to monitor and supervise the quality of mHealth APPs and wearable medical equipment. In this way, feedback, complaining, information coordination, and data release can be realized freely, and APPs with risks can be identified.

## 6. Limitations

This work has some limitations. Although the current testing flowchart aimed to maximize accuracy and objectivity, the research effectiveness might be limited from the following aspects. This work is based on 73 product samples and adopts usefulness indices for the first time for a systematic quantitative investigation into hypertension management and control mHealth APPs, which are products from a vertical subdivided domain. This investigation reveals the cross-sectional snapshot of the mHealth industry in mainland China in October 2016. First, the acquired APPs are small in number and target at the management of hypertension, so we are unable to completely explain the behaviors of the mHealth APP market. Second, this work was limited to non-HCP users and excluded HCP users. These two types of APP users are completely different in nature. In addition, paid APPs were excluded. The above reasons might have led to deviation in the analytical results.

## 7. Research Directions and Developing Trends

Future research trends should include medical institutions, HCP, and governmental duty offices. Therefore, service systems, laws and regulations, and business-profiting modes should be comprehensively analyzed from higher levels. More comprehensive sample subdivision should be studied, targeting personalized usability research involving the demands of users with different age groups, cultures, and habits. Moreover, problems regarding the supervision and management, security, privacy, and reliability of mHealth APPs should be solved as soon as possible.

## Figures and Tables

**Table 1:** Inclusion and exclusion criteria of target APPs.

Criterion name	Declaration
Inclusion criterion 1 (IC1)	The search terms were “高血压” or “hypertension.”
Inclusion criterion 2 (IC2)	Free Android or iOS APPs (paid APPs were excluded). (If one APP had both free and paid versions, the paid version was excluded.)
Inclusion criterion 3 (IC3)	Belonging to either health or medical APPs.
Inclusion criterion 4 (IC4)	Must include APPs for the hypertension prevention and treatment business.
Inclusion criterion 5 (IC5)	The target users were marked as mHealth APPs for non-health care professionals (non-HCP). HCPs referred to those included in the World Health Organization's health professional categorization [28].
Exclusion criterion1 (EC1)	APPs were only data receiving and transferring ends of external facilities or sensors and did not function for the prevention, treatment, and management of hypertension.
Exclusion criterion2 (EC2)	APPs did not support Chinese characters (either simple or traditional Chinese).

**Table 2:** Principles for PIPOH-based customization of hypertension management service factors.

Name	Description
Patients	Hypertensive patients
Major interventions	Including patient's awareness and recording of illness situation, data display [24], acquisition of knowledge about hypertension prevention and treatment [25], daily self-management, doctor-patient communication, between-patient experience exchange [26], references and foundation provided to doctors for diagnosis before clinical treatment [27], and other necessary assistance provided to patients
Target professional users of APPs	Non-HCP [28], nonhospital professionals defined by WHO
Therapeutic outcome	Hypertension prevention and treatment
Use environment	Daily life, nonclinic

**Table 3:** Service factor catalog of mHealth APPs.

Previously defined catalogue	Newly adjusted catalogue
Reminder	Calendar-based reminding
Telemedicine	Appointed registration and remote video consultation
Record	Hypertension prevention and control information records
Treatment	Drug use records
Patient monitoring	Automatic and/or manual data processing
Discussion	Communication and social networks
Medicine propaganda and education and literatures	Popularization, propaganda, and education
Call center	Localization service
Others	Others (emergency contact, time axis-based data display, data security, and privacy protection)

**Table 4:** The smallest usefulness evaluation item template of APPs.

Catalogue	China hypertension prevention and treatment mHealth service factor catalogue	Number	Evaluation items	ID
APP service evaluation	Calendar-based reminder	1	Does it have the function of calendar-based hospital or community treatment and management of chronic disease?	C1
		2	Does it have the function of calendar-based to-do list (e.g., drug use on that day or reminder, blood pressure measurement at preset time and reminder, and exercise event and reminder)?	C2
		3	Does it have the function of calendar-based remark? (Record some subjective symptom or remark information.)	C3
	Appointed registration	4	Does it have the function of extra bills for direct contact with doctors or online hospital registration?	A3
	Automatic and/or manual data processing	5	Does it have the function for the patient to manually input the necessary data above?	P1
		6	Does it support the acquisition of blood pressure and heart rate by using externally placed or inner sensors (e.g., acquisition of blood pressure and heart rate by using iHealth band or Xiaomi band; check list results; and medical records, pictures, or symptoms were photographed by cam)?	P2
	Communication and social networks	7	Does it integrate common Chinese social software such as WeChat, Weibo, or QQ?	S2
		8	Does it have the function to share information and communicate with other users (e.g., providing a module for patient community discussion, for discussing experiences, or free propaganda and education activities in community medical institutions)?	S3
	Popularization, propaganda, and education	9	Does it push or carry propaganda and recommendations on health habits?	A1
		10	Does it push or carry propaganda and recommendations on nutrition meals?	A2
	Localization service	11	Does it have the localization function that helps to localize patients or informed the patients about the position of the nearest doctor?	S1
	Others (emergency contact)	12	Does it have the function for setting emergency contact persons? Does it allow saving telephone and/or WeChat of contact persons?	E1
		13	Does it have the function of emergency contact, allowing to directly call the emergency contact persons via telephone and/or WeChat?	E2
		14	Does it have the function of urgency display page (Automatically displaying the abstract of patient's blood pressure and illness situation and emergency contact persons)? Does it allow visiting the doctor upon emergency treatment at convenience?	E3
	Others (time axis-based data display)	15	Does it have two basic timestamps of the patient's medical data (data generation timestamp and data record timestamp)?	T1
		16	Does it have the function of abstraction (automatic frequency reduction for the collected high-frequency data) and visualization (graph-like description of the patient's data) of data from spatial-temporal perspectives?	T2
	Others (data security and privacy protection)	17	Does the software allow offline input, acquisition, and use of data (cached locally and automatic synchronization upon loading)?	F1
		18	Does it have the function of single- or multiple-user authentication and authorization?	U1
		19	Does it have the function of urgent information acquisition?	U2
		20	Does it have the function of local storage and caching of data encryption?	U3
		21	Does it have the function of data signature antitampering?	U4
		22	Does it have the function of data backup?	U5
Data element evaluation	Hypertension prevention and control information records	23	History of present illness	D1
		24	Previous history	D2
		25	History of surgery	D3
		26	Social history (including smoking, alcohols, privacy, and occupation)	D4
		27	Family history	D5
		28	History of allergy	D6
		29	Recording of immunity and inoculation	D7
		30	Follow-up records (including one-to-one paired community doctor)	D8
		31	Laboratory examination results	D9
		32	Vital signs (height, weight, BMI, and blood pressure)	D11
		33	Demographic information of users (name, gender)	D12
	Drug use information records	34	Records of drug use situations	D10

**Table 5:** Selected details of APPs.

Number	APP name	Link to APP (accessed by 31 Oct. 2016)	Platform
1	xue-ya-guan-jia-gao-xue-ya	https://itunes.apple.com/cn/app/xue-ya-guan-jia-gao-xue-ya/id702674599?mt=8	iOS
2	gao-xue-ya-zhi-liao-mi-ji	https://itunes.apple.com/cn/app/gao-xue-ya-zhi-liao-mi-ji/id1114253242?mt=8	iOS
3	ti-jian-bao-ce-xue-ya-xin	https://itunes.apple.com/cn/app/ti-jian-bao-ce-xue-ya-xin/id1062204827?mt=8	iOS
4	kang-kang-xue-ya-gao-xue-ya	https://itunes.apple.com/cn/app/kang-kang-xue-ya-gao-xue-ya/id901362833?mt=8	iOS
5	gao-xue-ya-guan-jia	https://itunes.apple.com/cn/app/gao-xue-ya-guan-jia/id929001721?mt=8	iOS
6	tu-huan-jian-kang-nin-jia	https://itunes.apple.com/cn/app/tu-huan-jian-kang-nin-jia/id1117812930?mt=8	iOS
7	zhang-kong-gao-xue-ya	https://itunes.apple.com/cn/app/zhang-kong-gao-xue-ya/id740362713?mt=8	iOS
8	gao-xue-ya-zhi-liao-guan-jia	https://itunes.apple.com/cn/app/gao-xue-ya-zhi-liao-guan-jia/id1123479879?mt=8	iOS
9	xue-ya-xue-zhi-bao-jian-guan	https://itunes.apple.com/cn/app/xue-ya-xue-zhi-bao-jian-guan/id1039779655?mt=8	iOS
10	yue-tang-jian-kang-you-hua	https://itunes.apple.com/cn/app/yue-tang-jian-kang-you-hua/id984660846?mt=8	iOS
11	xue-ya-zhun-xiao-zhun-xue	https://itunes.apple.com/cn/app/xue-ya-zhun-xiao-zhun-xue/id1053971682?mt=8	iOS
12	gao-xue-ya-kang-fu-bao-dian	https://itunes.apple.com/cn/app/gao-xue-ya-kang-fu-bao-dian/id1020760423?mt=8	iOS
13	xun-yi-wen-yao-mian-fei-yi	https://itunes.apple.com/cn/app/xun-yi-wen-yao-mian-fei-yi/id586157918?mt=8	iOS
14	gao-xue-ya-zhi-nan-gao-xue	https://itunes.apple.com/cn/app/gao-xue-ya-zhi-nan-gao-xue/id1123479947?mt=8	iOS
15	xue-ya-diao-yang-ke	https://itunes.apple.com/cn/app/xue-ya-diao-yang-ke/id1147080258?mt=8	iOS
16	gao-xue-ya-zhi-duo-shao-gao	https://itunes.apple.com/cn/app/gao-xue-ya-zhi-duo-shao-gao/id978579995?mt=8	iOS
17	kang-kang-xue-ya-lian-tong-ban	https://itunes.apple.com/cn/app/kang-kang-xue-ya-lian-tong-ban/id957638415?mt=8	iOS
18	xue-ya-guan-jia	https://itunes.apple.com/cn/app/xue-ya-guan-jia/id740435884?mt=8	iOS
19	lao-nian-yang-sheng-man-xing	https://itunes.apple.com/cn/app/lao-nian-yang-sheng-man-xing/id1035227589?mt=8	iOS
20	tian-tian-xue-ya	https://itunes.apple.com/cn/app/tian-tian-xue-ya/id957664096?mt=8	iOS
21	ban-ge-yi-sheng	https://itunes.apple.com/cn/app/ban-ge-yi-sheng/id949812115?mt=8	iOS
22	jian-kang-yang-sheng-jian	https://itunes.apple.com/cn/app/jian-kang-yang-sheng-jian/id741986807?mt=8	iOS
23	yi-xue-xiao-gong-ju-zui-zhi	https://itunes.apple.com/cn/app/yi-xue-xiao-gong-ju-zui-zhi/id474137855?mt=8	iOS
24	yi-fang-jian-kang-ri-ji-zui	https://itunes.apple.com/cn/app/yi-fang-jian-kang-ri-ji-zui/id1005493950?mt=8	iOS
25	ji-shi-xin-lu-xin-zang-jian	https://itunes.apple.com/cn/app/ji-shi-xin-lu-xin-zang-jian/id409625068?mt=8	iOS
26	tai-guan-jia-jian-kang-zi	https://itunes.apple.com/cn/app/tai-guan-jia-jian-kang-zi/id1091138728?mt=8	iOS
27	xi-meng-jian-kang	https://itunes.apple.com/cn/app/xi-meng-jian-kang/id927853584?mt=8	iOS
28	zhang-shang-yi-sheng-zhang	https://itunes.apple.com/cn/app/zhang-shang-yi-sheng-zhang/id1104230972?mt=8	iOS
29	jin-dian-xue-ya-guan-li	https://itunes.apple.com/cn/app/jin-dian-xue-ya-guan-li/id898638656?mt=8	iOS
30	xue-ya-smart-xue-ya-smartbp	https://itunes.apple.com/cn/app/xue-ya-smart-xue-ya-smartbp/id519076558?mt=8	iOS
31	jian-kang998-wen-yi-sheng	https://itunes.apple.com/cn/app/jian-kang998-wen-yi-sheng/id1033474431?mt=8	iOS
32	wei-xun-yi-dong-yi-liao-gua	https://itunes.apple.com/cn/app/wei-xun-yi-dong-yi-liao-gua/id790208995?mt=8	iOS
33	yi-sheng-shu-zai-xian-wen	https://itunes.apple.com/cn/app/yi-sheng-shu-zai-xian-wen/id922382896?mt=8	iOS
34	kuai-su-wen-yi-sheng-guo-nei	https://itunes.apple.com/cn/app/kuai-su-wen-yi-sheng-guo-nei/id521634552?mt=8	iOS
35	dong-ri-zhong-yi	https://itunes.apple.com/cn/app/dong-ri-zhong-yi/id997661048?mt=8	iOS
36	runtastic-heart-rate-xin-lu	https://itunes.apple.com/cn/app/runtastic-heart-rate-xin-lu/id583311988?mt=8	iOS
37	hao-da-fu-zai-xian-zi-xun	https://itunes.apple.com/cn/app/hao-da-fu-zai-xian-zi-xun/id919502358?mt=8	iOS
38	gao-xue-ya	https://itunes.apple.com/cn/app/gao-xue-ya/id909967547?mt=8	iOS
39	guan-jia-yi-sheng	https://itunes.apple.com/cn/app/guan-jia-yi-sheng/id882701452?mt=8	iOS
40	zhong-yi-zhi-liao-yang-sheng	https://itunes.apple.com/cn/app/zhong-yi-zhi-liao-yang-sheng/id1038340460?mt=8	iOS
41	zhong-yi-jian-kang-xue-wei	https://itunes.apple.com/cn/app/zhong-yi-jian-kang-xue-wei/id1043463327?mt=8	iOS
42	mu-biao-jian-kang-wo-hu-lian	https://itunes.apple.com/cn/app/mu-biao-jian-kang-wo-hu-lian/id477774973?mt=8	iOS
43	sheng-ming-shu-xue-ya-tong	https://itunes.apple.com/cn/app/sheng-ming-shu-xue-ya-tong/id1180490472?mt=8	iOS
44	jian-kang-zhong-xin	https://itunes.apple.com/cn/app/jian-kang-zhong-xin/id698833425?mt=8	iOS
45	xi-en-jian-kang-zai-xian-wen	https://itunes.apple.com/cn/app/xi-en-jian-kang-zai-xian-wen/id1052965802?mt=8	iOS
46	jian-ya-bao	https://itunes.apple.com/cn/app/jian-ya-bao/id1027250761?mt=8	iOS
47	ifora-mp	https://itunes.apple.com/cn/app/ifora-mp/id834184294?mt=8	iOS
48	control-tension	https://itunes.apple.com/cn/app/control-tension/id721696484?mt=8	iOS
49	accutension	https://itunes.apple.com/cn/app/accutension/id1067641040?mt=8	iOS
50	dooland-health-bpmanager	http://www.webcitation.org/6xCa4YqBt	Android
51	supertw-apppj-bp	http://www.webcitation.org/6xCaKiKTO	Android
52	blt-bp	http://www.webcitation.org/6xCaPOalz	Android
53	bloodpressurelog	http://www.webcitation.org/6xCaRlZxF	Android
54	iBP Monitor	http://www.webcitation.org/6xCaWCwSc	Android
55	weightcaloriewatch	http://www.webcitation.org/6xCaix95N	Android
56	trackermonitor	http://www.webcitation.org/6xCakIasU	Android
57	feelymos-bluebp	http://www.webcitation.org/6xCalP6JE	Android
58	actionbloodpressure	http://www.webcitation.org/6xCarnW7T	Android
59	hj-healthcare	http://www.webcitation.org/6xCauWalE	Android
60	smartbloodpressure	http://www.webcitation.org/6xCayhkxh	Android
61	kang-hypertension	http://www.webcitation.org/6xCazfzc0	Android
62	freshware-bloodpressure	http://www.webcitation.org/6xCb0kuIg	Android
63	Bpservier	http://www.webcitation.org/6xCb5j9dy	Android
64	ffree-BloodPressure	http://www.webcitation.org/6xCb6mEyO	Android
65	lite-bptracker	http://www.webcitation.org/6xCbQpIuT	Android
66	cchong-BloodPressure	http://www.webcitation.org/6xCbRil0d	Android
67	openit-bpdiary	http://www.webcitation.org/6xCbUNW1L	Android
68	bpressure	http://www.webcitation.org/6xCbXgAk9	Android
69	bpbuster	http://www.webcitation.org/6xCbozy42	Android
70	jiang-kang-miao-guan-jia	http://www.webcitation.org/6xCbphofx	Android
71	HealthCheck	http://www.webcitation.org/6xCbqROiD	Android
72	mengtaoye-mybloodpressure	http://www.webcitation.org/6xCbr6SCC	Android
73	cardiojournal	http://www.webcitation.org/6xCc4gCYU	Android
